# Preparation and Characterization of Novel PBAE/PLGA Polymer Blend Microparticles for DNA Vaccine Delivery

**DOI:** 10.1155/2014/385135

**Published:** 2014-10-27

**Authors:** Meenashi Vanathi Balashanmugam, Sivagurunathan Nagarethinam, Hitesh Jagani, Venkata Rao Josyula, Abdulmohsen Alrohaimi, Nayanabhirama Udupa

**Affiliations:** ^1^Department of Pharmaceutical Biotechnology, Manipal College of Pharmaceutical Sciences, Manipal University, Manipal 576104, India; ^2^College of Pharmacy-Boys, Shaqra University, Al Dawadmi Campus, Al Dawadmi 11911, Saudi Arabia; ^3^Directorate of Research, Manipal University, Manipal 576104, India

## Abstract

*Context*. Poly(beta-amino ester) (PBAE) with its pH sensitiveness and Poly(lactic-co-glycolic acid) (PLGA) with huge DNA cargo capacity in combination prove to be highly efficient as DNA delivery system. *Objective*. To study the effectiveness of novel synthesized PBAE polymer with PLGA blend at different ratios in DNA vaccine delivery. *Methods*. In the present study, multifunctional polymer blend microparticles using a combination of PLGA and novel PBAE polymers A1 (bis(3-(propionyloxy)propyl)3,3′-(propane-1,3-diyl-bis(methylazanediyl))dipropanoate) and A2 (bis(4-(propionyloxy)butyl)3,3′-(ethane-1,2-diyl-bis(isopropylazanediyl))dipropanoate) at different ratios (85 : 15, 75 : 25, and 50 : 50) were prepared by double emulsion solvent removal method. The microparticles were characterized for cytotoxicity, transfection efficiency, and DNA encapsulation efficiency. *Result*. It was evident from results that among the microparticles prepared with PLGA/PBAE blend the PLGA : PBAE at 85 : 15 ratio was found to be more effective combination than the microparticles prepared with PLGA alone in terms of transfection efficiency and better DNA integrity. Microparticles made of PLGA and PBAE A1 at 85 : 15 ratio, respectively, were found to be less toxic when compared with microparticles prepared with A2 polymer. *Conclusion*. The results encourage the use of the synthesized PBAE polymer in combination with PLGA as an effective gene delivery system.

## 1. Introduction

The major limitation with genetic vaccines is lack of safe and efficient DNA delivery systems. Existing vaccine delivery systems, namely, viral, bacterial, synthetic, or physical delivery systems, are either unsafe or inefficient. This necessitated for safe and efficient delivery systems for successful gene therapy. In comparison to other synthetic delivery systems, polymeric delivery systems are better as they are safe and efficient due to their biodegradability and biocompatibility [1].

Particulate delivery system with Poly(lactic-co-glycolic acid) (PLGA), a Food and Drug Administration (FDA) approved polymer, has high potential in gene delivery. The main drawback associated with PLGA particles is the delayed* in vivo* delivery [2]. Additionally, substantial damage to plasmid DNA occurs because of acidic microclimate inside the particles [3].

On the other hand, poly(beta-amino ester) (PBAE) microparticles are pH responsive and effective at endosomal (acidic) delivery of plasmid DNA (pDNA) [4, 5] but are less immunogenic. The polymer is biodegradable and the biodegradation products formed such as *β*-amino acid are not harmful to the body [6, 7].

As research on biological delivery systems has progressed, comprehensive strategies such as the use of multifunctional delivery systems have emerged. Through a synergistic effect, multifunctional carriers are capable of overcoming distinct physiological barriers and delivering therapeutic payload(s) to the target site [8–10]. Safe and efficient delivery of pDNA by the synthetic polymer blend microparticles is the interest of today. Moreover, the effectiveness of nonviral delivery systems can be enhanced by use of adjuvants, cytokines, and self-replicating RNA systems [11–15].

In the present study, microparticles were prepared with polymer blend of PLGA and a novel PBAE polymer, synthesized in our lab. Evaluation of the microparticle formulation was carried out to know their potential as DNA delivery system.

## 2. Materials and Methods

### 2.1. Materials

PLGA 50 : 50 RESOMER (RG502 H) polymer was purchased from Evonik Röhm Pharma GmbH, Germany. PBAE polymers A1 (bis(3-(propionyloxy)propyl)3,3′-(propane-1,3-diyl-bis(methylazanediyl))dipropanoate) and A2 (bis(4-(propionyloxy)butyl)3,3′-(ethane-1,2-diyl-bis(isopropylazanediyl))dipropanoate) were synthesized in our lab. Plasmid encoding firefly luciferase (pCMV-Luc) was procured from ELIM Biopharmaceuticals, USA, and propagated in large quantity in competent* E. coli DH5α* cells. Further the plasmid was isolated and purified using mini preparation kit from Sigma chemical Co, (St. Louis, MO, USA). Bright glow luciferase assay system was procured from Promega, USA. Quant-iT PicoGreen dsDNA Reagent and Kits were procured from Invitrogen Life Technologies Co. (USA). 3-(4,5 dimethyl thiazole-2 yl)-2,5-diphenyl tetrazolium bromide (MTT) from Sigma chemical Co, St. Louis, MO, USA was used in studies. Dimethylsulfoxide (DMSO), isopropanol were procured from Qualigens fine chemicals, Mumbai, India. Trypsin, 4-(2-hydroxyethyl)-1-Piperazineethanesulphonic acid (HEPES) sodium salt buffer saline, and normal melting agarose were procured from Himedia lab Pvt. Ltd., Mumbai, India. Lipofectamine 2000 and fetal bovine serum (FBS) were purchased from life technologies, Carlsbad, USA. All other chemicals used in the study were of analytical grade.

### 2.2. Cell Lines

EL4 (murine thymoma cancer) cell line was procured from National Centre for Cell Science (NCCS), Pune, India, and cultured in Dulbecco's modified Eagle's medium (DMEM) from Sigma, USA, supplemented with 10% FBS and 1% penicillin/streptomycin from Himedia, Mumbai, and incubated at 37°C in 5% CO_2_ humidified atmosphere.

### 2.3. Formulation of Polymer Blend Microparticles

pDNA-loaded PLGA/PBAE microparticle formulations were prepared by the water-in-oil-in-water (w/o/w) double emulsion solvent removal (DESR) method. Microparticles of PLGA and PBAE polymer blend were prepared at different ratios, namely, 85 : 15, 75 : 25, 50 : 50 using dichloromethane (DCM) as solvent. Aqueous pDNA (pCMV-Luc) solution was prepared with (1 mM) EDTA and (300 mM) D (+) lactose. The ratio of pDNA to polymer was 1 : 200. The plasmid solution was added into the polymer solution and sonicated for 30 s using a probe sonicator (Sonics and Materials Inc., USA) at 60% amplitude. The resulting emulsion was immediately added to a homogenized solution of 0.5% W/V polyvinyl alcohol (PVA) solution and homogenized at 12000 rpm using hand homogenizer (polytron PT-1300D, Thermo Fisher Scientific Inc., USA) for 3 min on ice bath. The final w/o/w emulsion was stirred for 45 min on ice bath. The microparticles were centrifuged at 7500 rpm for 10 min at 4°C and washed to remove excess of PVA prior to lyophilization. The microparticles were lyophilized with cryoprotectant 5% mannitol (1 : 1 ratio) and stored in −20°C until further use.

### 2.4. Characterization of Polymer-Blend Microparticles

#### 2.4.1. Particle Size, Zeta Potential, Surface Morphology, and DNA Encapsulation

Particle size and zeta potential were determined using Malvern Nano Zetasizer, (Malvern Instruments Ltd., Worcestershire, UK). Malverns DTS v.5.00 software (Malvern Instruments Ltd., Worcestershire, UK) was used for data acquisition.

Surface morphology of prepared microparticles was determined using scanning electron microscopy (SEM) with EDS (JEOL, Akishima, Tokyo, Japan). pDNA encapsulation efficiency was determined by an indirect method (i.e.) indirectly measuring the amount of pDNA present in the supernatant after recovering the microparticles. Briefly, the primary formulation was centrifuged and microparticles were recovered as pellet. The amount of pDNA present in the supernatant (DNA_supernat_) was estimated using PicoGreen assay method in a black 96-well plate using fluorescent plate reader FLx800 from Biotek using Quant-iT PicoGreen dsDNA Reagent and Kits [16]. The amount of pDNA encapsulated (DNA_encap_) was calculated by subtracting the amount of pDNA in the supernatant (DNA_supernat_) from total amount of pDNA (DNA_total_) used for formulation.

#### 2.4.2. DNA Release Profile

For* in vitro *release profile study, the microparticles were incubated in 1 ml saline buffer (pH 7.4) and stirred using magnetic stirrer at 50 rpm. During sample withdrawal at each time point, the samples were centrifuged at 10,000 rpm and the supernatant was collected. Supernatants were withdrawn at different intervals, namely, 24, 48, 72, 96 h, and fresh saline of equal volume was replaced to maintain constant volume. The amount of pDNA present in the supernatant was estimated using PicoGreen assay kit in a black 96-well plate using fluorescent plate reader FLx800 from Biotek [16, 17].

#### 2.4.3. DNA Integrity

The integrity of the encapsulated pDNA was determined by gel electrophoresis using 1% agarose gel containing ethidium bromide. Gel electrophoresis was carried out with 1x tris acetate EDTA (TAE) buffer at a constant voltage of 60 V for 45 min. After electrophoresis, pDNA in the gels was visualized using Alpha Imager gel documentation system from AlphaInnotech (multi-image light cabinet). pDNA obtained from supernatants, after incubation of the microparticles in saline for 24–96 h, was pooled up and used for evaluating the pDNA integrity. The integrity of released plasmid was compared with that of the naked plasmid.

### 2.5. *In Vitro* Studies

#### 2.5.1. Cytotoxicity

*In vitro* cytotoxicity of microparticles was evaluated by MTT assay using* EL4* cell line. Assay was performed for a time interval of 48 h and the results were documented using ELISA plate reader ELx800  MS from Biotek [18].

#### 2.5.2. Transfection Efficiency

The transfection ability of the pCMV-Luc microparticles on* EL4* cells was evaluated using bright glow luciferase assay system (Promega, USA) in a white 96-well plate. Lipofectamine 2000 was used as standard and the relative transfection efficiency of the formulation was determined using fluorescent plate reader FLx800 from Biotek [7].

### 2.6. Statistical Analysis

All experiments were carried out in triplicate and the data were reported as mean ± standard deviation and/or standard error.

## 3. Results

### 3.1. Formulation and Characterization of Polymer Blend Microparticles

Microparticles, comprising a blend of PBAE and PLGA polymers, were prepared by DESR method. The mean particle size of prepared microparticles was in the range of 0.79 to 1.88 *µ*m and the zeta potential was in the range of −15 to −38.1 mV (Table 1). In case of PLGA : A2 microparticles, we could observe a decrease in negativity and shift towards the positive side as the concentration of A2 polymer increased in the formulation, whereas, in case of PLGA : A1 microparticles, such linearity was not observed. It was observed that the size of the microparticles prepared with PLGA alone was smaller when compared with PLGA/PBAE blend. Encapsulation efficiency was found to be higher in PLGA/PBAE microparticles compared to PLGA alone. Moreover, with increase in PBAE content in microparticles, the encapsulation efficiency was increased from 10 to 15% (Table 1). PLGA : A1 microparticles showed better encapsulation efficiency when compared with PLGA : A2 microparticles with a difference of around 5–7%.

### 3.2. Surface Morphology of the Microparticles by SEM

**Figure 1:**
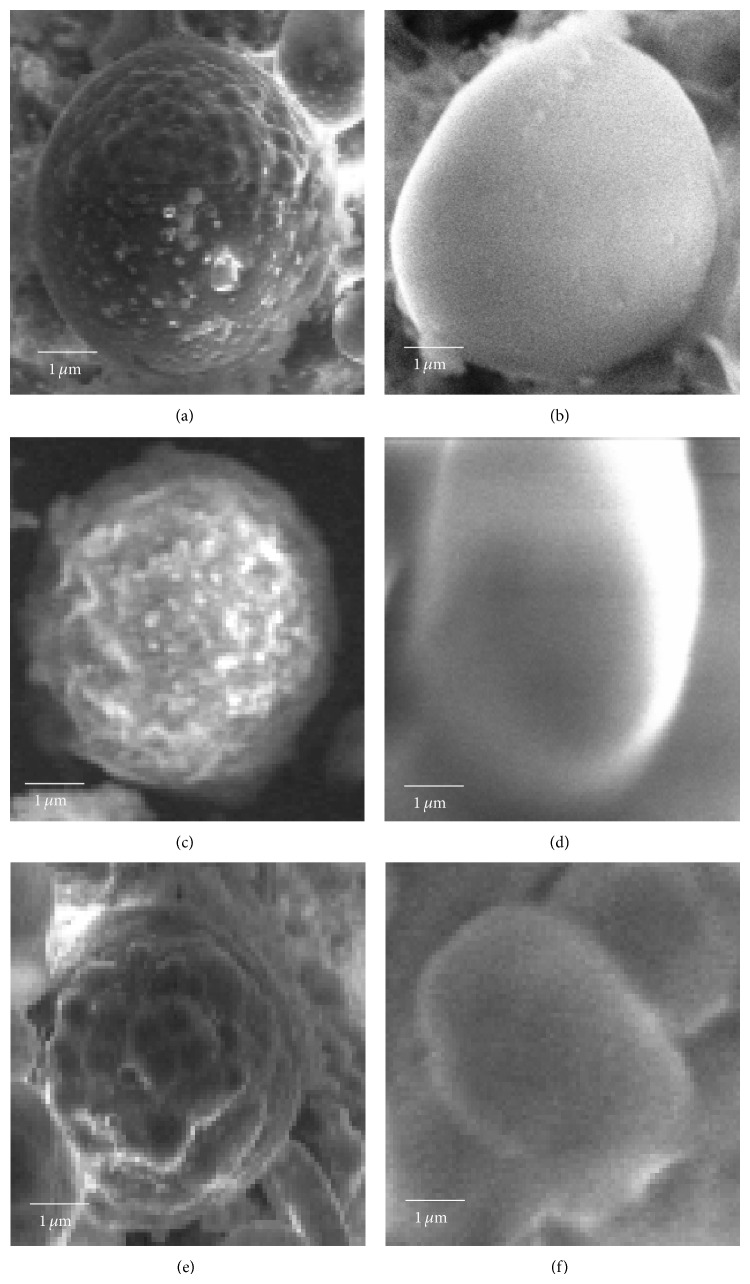
Scanning electron microscopy of microparticles. (a) PLGA : A1 (85 : 15), (b) PLGA :  A2 (85 : 15), (c) PLGA : A1 (75 : 25), (d) PLGA : A2 (75 : 25), (e) PLGA : A1 (50 : 50), and (f) PLGA : A2 (50 : 50).

Surface morphology of the microparticles was studied by scanning electron microscopy (Figure 1). With increased PLGA content, the surface of microparticles appeared smoother. Microparticles, prepared with A1 polymer, were more spherical and uniform compared to microparticles prepared from A2 polymer. The surface of microparticles prepared from A2 polymer was smoother than that of microparticles prepared from A1 polymer.

### 3.3. pDNA Release Profile

**Figure 2:**
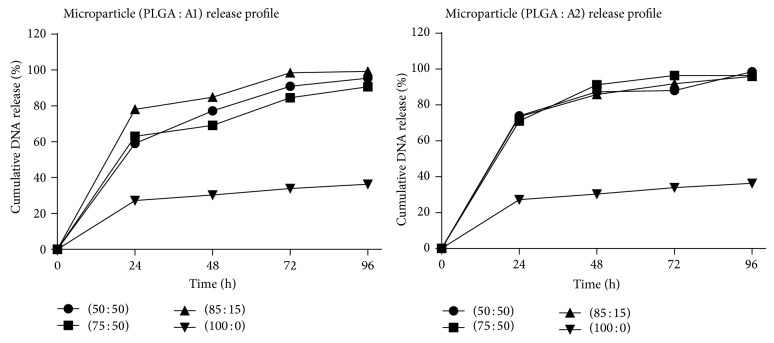
DNA release profile from microparticles.

The amount of pDNA released at different time intervals was determined by PicoGreen assay. A1 and A2 polymer blend microparticles exhibited high initial burst phase with pDNA release above 50% within 24 h, whereas microparticles made of PLGA alone exhibited low initial burst with pDNA release less than 30% and showed a delayed release over time. With increase in PBAE content above 15%, the microparticles exhibited delayed release. Overall, within 96 h, nearly 90% of pDNA was released from polymer blend microparticles, whereas only 36% pDNA was released from microparticles prepared with PLGA alone (Figure 2).

### 3.4. pDNA Integrity

**Figure 3:**
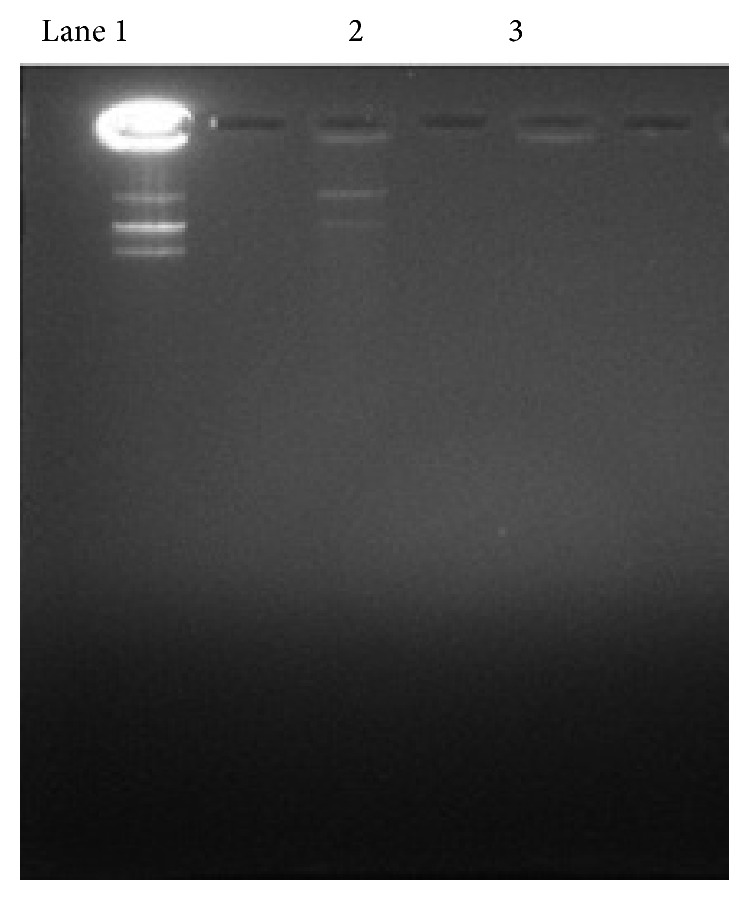
DNA integrity study by gel retardation assay. Lane 1: pDNA control and Lane 2: PLGA : A1(85 : 15) 3-PLGA alone.

The integrity of released pDNA was assessed using agarose gel electrophoresis. Integrity of pDNA from polymer blend microparticles was substantially higher than that from PLGA microparticles. In samples from polymer blend microparticles, three intact bands of pDNA were observed similar to that of the untreated plasmid control, whereas in case of samples from PLGA microparticles, no clear bands were observed in the run lane; instead a band was observed in the well alone (Figure 3).

### 3.5. Cytotoxicity

**Figure 4:**
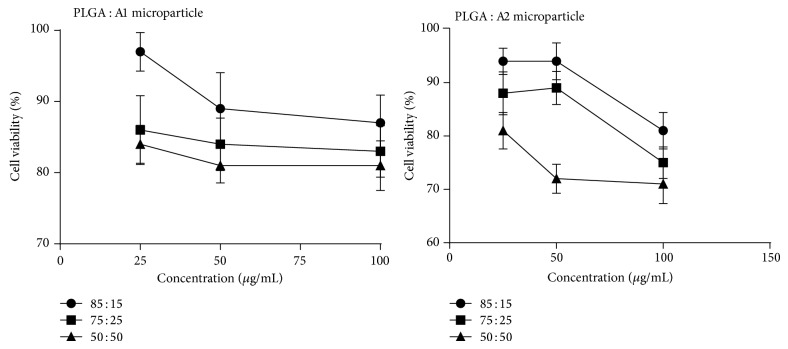
Cytotoxicity of microparticles on EL-4 cells by MTT assay.

Toxicity of the microparticle formulations on EL4 cells was determined by MTT assay (Figure 4). More than 70% of cells were found to be viable after treatment with formulations. With increase in PBAE content, there was a slight decrease in cell viability. Microparticles made of PLGA and A1 polymer (85 : 15) were less toxic even at high concentration with minimum cell viability of 87%. Moreover, microparticles made of A1 polymer were less toxic when compared to A2 microparticles. PLGA and PBAE polymer were found to be nontoxic at tested concentration.

### 3.6. Transfection Efficiency

**Figure 5:**
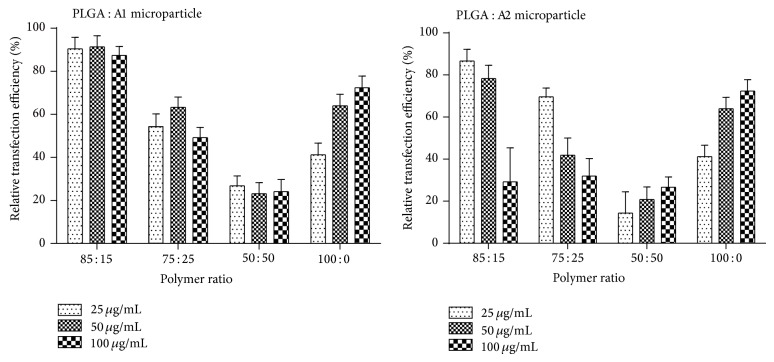
Relative transfection efficiency of microparticles by bright glow luciferase assay.

The microparticle formulations were tested for their ability to transfect EL4 cells by firefly luciferase assay. The relative transfection efficiency was calculated using lipofectamine 2000 complexed with pCMV-Luc plasmid as positive control (Figure 5). The relative transfection efficiency of the PLGA : A1 microparticles at 85 : 15 ratio was found to be more than 85% at all tested concentrations. Among polymer blend microparticles formulations, except in the case of PLGA : A2 (50 : 50), the transfection efficiency decreased with increased dose of microparticles. Among the polymer blend microparticles, with increase in PBAE content, the transfection efficiency was decreased. Transfection efficiency was found to be the least with PLGA : A2 microparticles at 50 : 50 ratio. In case of PLGA alone, the transfection efficiency was increased with increase in the concentration of microparticles.

## 4. Discussion

Potency of genetic vaccine is a factor of gene expression levels. Hence, designing of DNA vaccine delivery system is to be done with an aim to deliver the DNA to the target cells and also to increase the levels of expression of the pDNA to evoke adequate immune response. Research has been progressing in the search for an efficient synthetic delivery system in order to decrease the dose of pDNA required to induce an immune response, to protect the pDNA from endonucleases and to bring about better transfection of the pDNA into the nucleus of the target cells. Today, we have many synthetic delivery systems, studied for their efficiency in gene delivery. Polymeric delivery systems have the advantages of protecting pDNA from endonuclease action and microparticle delivery systems are good at delivering their pDNA content to the target immune cells due to their size influencing factor [19]. Microparticles are better phagocytized by the immune cells rather than the nanoparticles as a factor of size [14, 15]. Microparticles prepared with PLGA were studied for vaccine delivery, but their use was limited due to damage to pDNA caused by the internal acidic microclimate and the less transfection efficiency [3, 14]. Poly(ethylene imine) (PEI) a gold standard polymer for good transfection efficiency was also studied for gene delivery to a greater extent, but severe cytotoxicity restricts its usage [20–22].

In our present study, we have used novel PBAE polymers synthesized in our lab. They are pH responsive, they undergo hydrolysis at extreme acidic or basic conditions due to which they are effective at endosomal (acidic) delivery of plasmid DNA, and their biodegradation products are safe, nontoxic to the cells. They have the ability to condense and protect the pDNA which leads to an increase in vaccine potency [7]. PLGA has a unique property to hold heavy payload of pDNA and was used in combination with our synthesized PBAE polymer at different ratios. The most effective combination of PLGA and PBAE was selected based on DNA encapsulation and transfection efficiencies.

**Figure 6:**
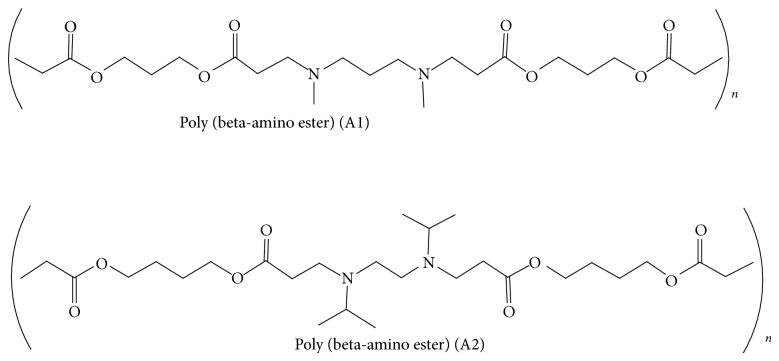


Among the different polymers synthesized in our lab, the A1 polymer and A2 polymer were selected due to their least cytotoxicity and better transfection efficiency. Polymers were synthesized by Michael addition reaction [23], where the nucleophilic addition reaction between the diamine and diacrylate at (1 : 1.2) ratio took place at the *β* unsaturated carbon atom to form poly(beta-amino esters). The structure of the polymers were confirmed by IR and NMR studies as given in Figure 6.

Using DESR method, the microparticles were successfully prepared with blend of PLGA and PBAE at different ratios (85 : 15, 75 : 25, 50 : 50) with size range of 0.8 to 1.9 *µ*m which was suitable for passive delivery into phagocytic cells, a prime need for DNA vaccine. The preparation of microparticles was optimized based on yield, particle size, DNA integrity, and dispersivity. The optimized conditions as given in the materials and methods section were used for preparation of microparticles. Dispersivity, one of the most important parameters, was greatly influenced by addition of surfactant and the solvent removal method from the formulation. Double emulsion solvent evaporation method gives rise to microparticles with very less dispersivity, and solvent removal method greatly improved the dispersivity of the formulation. The optimum concentration of surfactant (poloxamer F68) used in the formulation to improve the dispersivity was 25 mg/L. As the presence of surfactant in the formulation may influence the integrity of microparticles, care was taken to select concentration which was not cytotoxic to cell lines and at the same time does not damage the pDNA entrapped in the microparticle. Poloxamer was used as surfactant as it also increases gene expression [24].

We have compared the microparticles made out of PLGA and A1 polymer and A2 polymer at different ratios, namely, 85 : 15; 75 : 25; 50 : 50, using standard methods and compared the efficiency of the two polymers in DNA vaccine delivery.

The proportionate increase in encapsulation efficiency with increasing PBAE content in polymer blend microparticles may be correlated to the electrostatic interactions between pDNA and PBAE [25]. Moreover, this interaction could also be the reason for high DNA integrity observed with polymer blend microparticles. In case of PLGA : A2, a strong correlation of zeta potential, encapsulation efficiency, and PBAE content were observed. The interaction between PBAE and pDNA could have been responsible for the increase in zeta potential and encapsulation efficiency [14, 25, 26].

SEM analysis revealed an increase in smoothness of the microparticle surface morphology with increase in PLGA content. The rough surface observed with increase in PBAE content could be due to the difference in rate of evaporation of the common solvent from the polymers [27, 28]. In comparison the PLGA : A2 polymer blend gave rise to microparticles with a smooth surface when compared with PLGA : A1 polymer microparticles which had a number of pores on its surface, thus giving us a lead that the A1 polymer microparticles are best suited for surface modification studies [29].

The high initial burst exhibited by polymer blend microparticles could be attributed to the difference in aqueous solubility as among the polymers and due to formation of pores and cracks in the microparticle surface due to solvent evaporation [30–32]. A1 polymer is highly water soluble than A2 polymer and PLGA. However, the sustained release pattern observed with increased PBAE content could be because of binding of free PBAE with free DNA, which could have masked the detection of DNA or could have resulted in sustained release of DNA over time. Additionally, with A1 polymer, increase in PBAE content above 15% affected the release profile. This could have resulted because of the increased polymer-DNA interaction, which is evident from enhanced encapsulation of DNA [33]. The delayed release profile is a property of PLGA and hence maybe a lower release profile was exhibited by PLGA microparticles. This may be partially attributed to degradation of DNA inside acidic microclimate. Since DNA was degraded it could have gone undetected even upon swelling and pore formation.

DNA integrity was higher with polymer blend microparticle in comparison to PLGA alone. Degradation of DNA occurs because of the acidic microclimate existing inside PLGA particle as PLGA when placed in aqueous phase upon degradation produce acids. As suggested earlier [14], PBAE could buffer the acidic microclimate, thus protecting the DNA. Moreover, the low levels of supercoiled DNA observed could be because of the intensive binding with PBAE, resulting in change in their conformation.

Transfection by polymer blend microparticles was high at 15% PBAE, whereas with increase in PBAE content, transfection efficiency was decreased. Moreover, a strong correlation exists between increase in PBAE content and toxicity. Additionally, at high concentration, polymer blend microparticles showed low transfection, whereas microparticles made of PLGA alone showed increase in transfection efficiency with increased concentration. This indicates that the decrease in transfection efficiency in polymer blend microparticle is because of toxicity associated with PBAE polymer as more amount of PBAE-pDNA complex enters the cell due to good transfection but results in cell death due to the high accumulation of PBAE, hence no expression of the reporter gene in the transfected cells [34]. However, inclusion of PBAE at lower concentration enhanced transfection which could be attributed to the DNA protective nature and increased encapsulation efficiency exhibited by polymer blend microparticles.

Thus, from the present study, it is evident that the microparticles made out of PLGA/PBAE blend and the PLGA : PBAE (85 : 15) ratio were found to be more effective combination than the microparticles made out of PLGA alone in terms of transfection efficiency and better DNA integrity.

## 5. Conclusion

In recent years, the research on synthetic delivery system for gene delivery has been carried out in many directions. The present development in DNA vaccine delivery is to use multifunctional polymer blend for preparation of particulate delivery system. The most important step is the selection of polymer combination to give a synergistic effect in terms of improved transfection efficiency and DNA integrity. Even though there are many polymeric systems available in the market, the most widely accepted and studied one is the FDA approved PLGA polymer. Hence in the present study, we have selected PLGA as one of the polymers and used newly synthesized PBAE polymer to overcome the drawbacks of PLGA, such as delayed* in vivo* delivery, damage to the pDNA due to internal acidic microclimate. In addition, the PBAE polymeric microparticles have the inherent ability to target the immune cells and their effective intracellular delivery has paved their way in DNA vaccine delivery system. There are studies done on PBAE microparticles containing the gene encoding for malignancies, but yet to be studied on infectious models. From the present study the adjuvancy and transfection efficiency associated with PBAE class of polymer along with PLGA were proved to be an effective combination for DNA vaccine delivery system. We have observed significant difference between the two different PBAE polymeric microparticle formulations, in terms of particle size, zeta potential, encapsulation efficiency, cytotoxicity, and transfection efficiency. Among the different ratios used in the study, we found that PLGA : A1 (85 : 15) was effective even at low concentration of the microparticles with least cytotoxicity. Further studies on enhancement of immunogenicity, targeting the immune cells using ligands, and studies involving* in vivo *immune challenge model are needed to confirm the results of our work.

## Figures and Tables

**Table 1:** Characterization of microparticles with respect to mean diameter, zeta potential, mean loading, and encapsulation efficiency.

Formulation	Mean diameter (nm)	Zeta potential (mV)	Mean loading (*µ*g/mg)	Encapsulation efficiency (%)
PLGA : A1(50 : 50)	1034 ± 22.4	−18.1 ± 1.67	1.98 ± 0.12	19.88 ± 1.2
PLGA : A1(75 : 25)	997 ± 12.5	−23.2 ± 1.5	1.49 ± 0.15	14.96 ± 1.55
PLGA : A1(85 : 15)	1882 ± 23.7	−17.5 ± 2.4	1.32 ± 0.07	13.29 ± 0.72
PLGA : A2(50 : 50)	821.2 ± 11.5	−15 ± 2.83	1.42 ± 0.09	14.28 ± 0.95
PLGA : A2(75 : 25)	1121 ± 20.1	−23.1 ± 1.09	1.14 ± 0.14	11.44 ± 1.48
PLGA : A2(85 : 15)	797.1 ± 9.88	−36.3 ± 2.47	0.98 ± 0.18	9.88 ± 1.8
PLGA alone (100 : 0)	612.9 ± 6.39	−38.1 ± 1.96	0.33 ± 0.04	3.325 ± 0.43

All the values are represented mean values of three independent experiments.
